# Dynamic Profiling and Prediction of Antibody Response to SARS-CoV-2 Booster-Inactivated Vaccines by Microsample-Driven Biosensor and Machine Learning

**DOI:** 10.3390/vaccines12040352

**Published:** 2024-03-25

**Authors:** Sumin Bian, Min Shang, Ying Tao, Pengbo Wang, Yankun Xu, Yao Wang, Zhida Shen, Mahamad Sawan

**Affiliations:** 1 CenBRAIN Neurotech Center of Excellence, School of Engineering, Westlake University, Hangzhou 310024, China; biansumin@westlake.edu.cn (S.B.);; 2 Department of Cardiology, Sir Run Run Shaw Hospital, School of Medicine, Zhejiang University, Hangzhou 310058, China; 3 Key Laboratory of Cardiovascular Intervention and Regenerative Medicine of Zhejiang Province, Hangzhou 310058, China

**Keywords:** microsampling, machine learning, neutralizing antibodies, inactivated vaccines, binding antibodies

## Abstract

Knowledge of the antibody response to the third dose of inactivated SARS-CoV-2 vaccines is crucial because it is the subject of one of the largest global vaccination programs. This study integrated microsampling with optical biosensors to profile neutralizing antibodies (NAbs) in fifteen vaccinated healthy donors, followed by the application of machine learning to predict antibody response at given timepoints. Over a nine-month duration, microsampling and venipuncture were conducted at seven individual timepoints. A refined iteration of a fiber optic biolayer interferometry (FO-BLI) biosensor was designed, enabling rapid multiplexed biosensing of the NAbs of both wild-type and Omicron SARS-CoV-2 variants in minutes. Findings revealed a strong correlation (Pearson r of 0.919, specificity of 100%) between wild-type variant NAb levels in microsamples and sera. Following the third dose, sera NAb levels of the wild-type variant increased 2.9-fold after seven days and 3.3-fold within a month, subsequently waning and becoming undetectable after three months. Considerable but incomplete evasion of the latest Omicron subvariants from booster vaccine-elicited NAbs was confirmed, although a higher number of binding antibodies (BAbs) was identified by another rapid FO-BLI biosensor in minutes. Significantly, FO-BLI highly correlated with a pseudovirus neutralization assay in identifying neutralizing capacities (Pearson r of 0.983). Additionally, machine learning demonstrated exceptional accuracy in predicting antibody levels, with an error level of <5% for both NAbs and BAbs across multiple timepoints. Microsample-driven biosensing enables individuals to access their results within hours of self-collection, while precise models could guide personalized vaccination strategies. The technology’s innate adaptability means it has the potential for effective translation in disease prevention and vaccine development.

## 1. Introduction

The antibody response to inactivated SARS-CoV-2 vaccines after the third dose (In-Vx-3), after two prime doses of inactivated vaccines, has not been well investigated in the general population. This knowledge is crucial, as the homologous whole inactivated vaccine booster is the primary vaccination program in China and many other countries. Given the possibility of recurring waves of COVID-19 outbreaks every six months, the potential for millions to be infected cannot be underestimated [1]. As we know, BA.4/5 and BF.7 were the dominant strains during the December 2022 infection wave in China, and XBB.1.5 is a highly infectious subvariant that drove a new wave of infections in early 2023. How the In-Vx-3 effects the latest Omicron variants is less well studied. Some studies have shown that the In-Vx-3 boosted the antibody response in individuals who had a low antibody response after the initial two doses and increased the level of neutralizing antibodies (NAbs) by up to five times in the general population [2,3]. More research is needed to evaluate the immune persistence of the homologous In-Vx-3 and the antibody response towards the latest variants.

NAb levels correlate with immune protection from the SARS-CoV-2 infection [4]. However, the standard NAb assays, including the pseudovirus neutralization test (PVNT) [5], have limitations because they require strict operating environments and well-trained personnel to perform complex procedures. To address these issues, we designed a rapid and automated fiber optic biolayer interferometry (FO-BLI) biosensor to detect NAbs in human sera, with clinical applicability [6]. Nonetheless, it is uncertain whether the NAb levels obtained through this biosensor precisely reflect the observed neutralization capacities of conventional assays. Moreover, venipuncture, a standard sampling method in hospitals, may not be ideal for frequent blood collection because it places additional burden and risk on healthcare systems and individuals. Meanwhile, microsampling has been established as a simple and painless method for antibody evaluation [7,8,9,10]. However, its utilization to evaluate NAbs is limited due to technical challenges, and clinicians continue to use traditional assays, which delays decision-making. Therefore, clinical practice requires a rapid biosensor that matches the performance of a standard assay for multiplexed biosensing of NAbs towards both the wild-type (WT) strain and latest subvariants of the COVID-19 virus. A microsample-driven biosensor will be particularly valuable during a pandemic due to its non-invasive, high-throughput characteristics and ability to reveal the vaccine’s efficacy. In addition to monitoring antibody levels, it is crucial to predict these levels before gathering sample measurements. This approach allows for individualized vaccination planning, which, in turn, facilitates the implementation of proactive measures to prevent potential outbreaks. From this perspective, machine learning (ML) has been used to predict the antibody response to the second and third doses of mRNA vaccines in transplant recipients [10,11]. However, both of these studies primarily focused on identifying the clinical predictors of the antibody response instead of predicting the antibody levels or their evolution over time in an individual.

To address these challenges, we integrated dried blood spot (DBS) microsampling with the multiplexed FO-BLI biosensor to dynamically profile NAb levels in 15 In-Vx-3 healthy donors. Subsequently, we used ML algorithms to predict the antibody levels for the same group of donors at various timepoints, using a dataset that included physiological parameters and optical measurements. Using the FO-BLI NAbs biosensor, we further evaluated the extent to which the latest variants escaped the In-Vx-3’s neutralizing antibodies against the wild-type variant (WT-NAbs). To gain a richer insight into the antibody response, we also developed a simplified FO-BLI biosensor for the multiplexed detection of the binding antibodies (BAbs) of different variant lineages. The identification of BAbs is also crucial for understanding antibody-dependent enhancement (ADE), a serious detrimental effect where the presence of BAbs post-vaccination may worsen subsequent viral infections in vitro and in vivo [12]. This represents a major impetus for the present study; a reliable and efficient diagnostic identification and quantification of NAbs and BAbs in patients’ blood. Originally planned to last three months after the booster dose, the study continued for six additional months, after which six donors provided samples on Day 270. This process enabled us to understand how the relaxed epidemic control measures, followed by the further outbreak, affected the antibody response. This study paves the way for an individualized vaccination strategy and improved public health decision-making. Also, the protocol for this integrated technology is well-established and highly adaptable, making it readily applicable for real-world uses in disease prevention and vaccine evaluation across various contexts.

## 2. Materials and Methods

### 2.1. Study Design

**Scheme 1:**
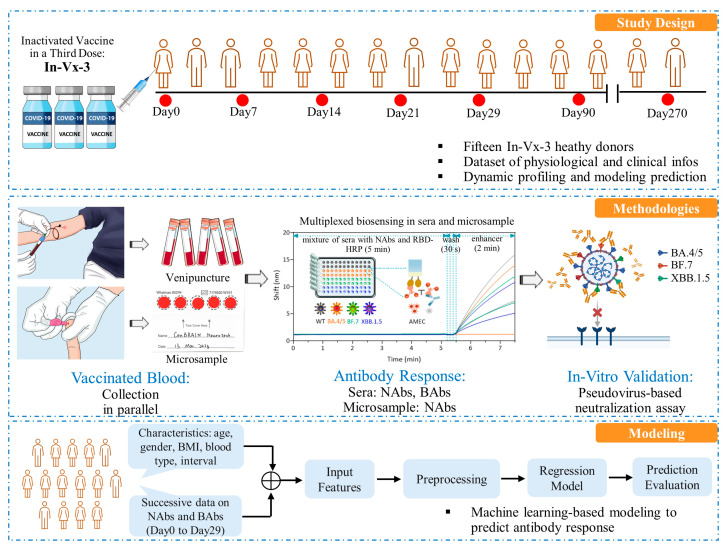
Scheme of the study design. The study involves recruiting fifteen healthy donors who received booster-inactivated vaccine, collecting blood in parallel, conducting the multiplexed biosensing of the antibody response, validating the results using an in vitro pseudovirus-based neutralization test, and applying ML to predict the antibody response in individuals.

The study design, the methodologies and the modeling used in this study for dynamic monitoring and precise prediction of antibody response to booster-inactivated vaccines is described in detail in Scheme 1. Notably, all volunteers received either Sinopharm or Sinovac as their original dose, with the third dose being either of them as well. Both inactivated vaccines utilized in this study are whole virus vaccines, administered according to the official guidance provided by the company at the local hospital.

### 2.2. Materials and Equipment

The proteins used were SARS-CoV-2 related proteins (Sino Biologicals, Beijing, China), including Biotinylated ACE2 Protein Human Recombinant, Spike RBD-His Recombinant Protein, BA.4/BA.5/BA.5.2 Spike RBD Protein, BA.4.6/BF.7 Spike RBD Protein, XBB Spike RBD Protein. The antibodies used included Spike Neutralizing Antibody-Rabbit Mab (Sino Biologicals, Beijing, China) and Research Grade Bebtelovimab-DVV00319 (AtaGenix, Wuhan, China). The kits used included Biotinylation Kit/Biotin Conjugation Kit (Fast, Type B); Lightning-Link^®^, HRP Conjugation Kit; Lightning-Link^®^ and Anti-Rheumatoid Factor IgM ELISA Kit (Abcam, Shanghai, China); and ImmPACT* AMEC Red* Substrate Kit (Vectorlabs, Shanghai, China). The agents used included Tween 80, Tween 20 and Bovine Serum Albumin (Sigma-Aldrich, Shanghai, China); SuperBlock™ (PBS) Blocking Buffer and SuperBlock™ T20 (PBS) Blocking Buffer (Thermo Scientific, Shanghai, China). The freshly prepared buffers included Sample diluent (SD) buffer [i.e., PBS (10 mM, pH 7.4) containing 0.02% Tween 20 and 0.1% BSA] and high-salt SD buffer (i.e., SD containing 274 mM NaCl). Other materials included Whatman^TM^ 903 Protein Saver Card (Cytiva, Shanghai, China); 96-well polystyrene black microplates (Greiner Bio-One GmbH, Shanghai, China); Streptavidin (Sartorius Group, Gottingen, Germany); and BD Microtainer^®^ contact-activated lancet (BD, Shanghai, China). The equipment used included an Octet^®^ K2 2-Channel System; a RED96E 8-Channel System (Sartorius Group, Gottingen, Germany); a Biotek ELx808 microplate reader (BioTek Instruments, Winooski, VT, USA); and an Axiocam 208 color (ZEISS, Oberkochen, Germany).

### 2.3. Designing the FO-BLI Biosensor for Multiplexed Detection of NAbs

The multiplexed NAbs FO-BLI biosensor was adapted from a previous version [6] with two major improvements. First, the optical signal was enhanced using a more environment- and user-friendly biomaterial 3-Amino-9-ethylcarbazole (AMEC). AMEC-based biosensing resulted in a higher signal-to-noise ratio and the precipitates generated were soluble in alcohols, providing an opportunity for fiber regeneration [13]. Second, a high-salt SD buffer was used to dilute the serum 100-fold to ensure minimal interference from blood, as proved in another recent study by us [14]. Similarly, the FO-BLI NAbs biosensor employed HRP-conjugated RBDs to compete with NAbs present in the sample. If the HRP-RBDs outcompete NAbs in either affinity or amount, AMEC will bind with HRP to produce a high number of precipitates, thereby amplifying the signals in fractions of seconds. In this study, NAbs were evaluated towards both WT and subvariants. Thus, WT-R001 continued to serve as the calibrator for WT-R001, while BEB was selected as the calibrator for Omicron NAbs. BEB is a potent and fully human NAb with broad neutralizing activity on SARS-CoV-2 variants of concern, including Omicron variant lineages [15].

### 2.4. Evaluating the Fibers’ Reproducibility for FO-BLI NAbs Biosensor

We assessed whether the precipitates induced by AMEC on the fiber tips could be entirely dissolved in ethanol, allowing for the fibers to be cleaned and regenerated. Evaluation was performed on 15 individual fibers, on which a mixture of a blank sample with HRP-WT was applied to interact with AMEC, resulting in final optical signals ranging from 0.5 to 40 nm, controlled by adjusting the reaction duration. The 15 precipitated fibers were divided into three groups. Group one was used to observe the surface morphology before any treatment, while groups two and three were immersed in high-purity 99.75% ethanol at 4 °C for 2 h and overnight, respectively, to demonstrate the changes in surface morphology after treatment.

### 2.5. Establishing DBS Method for NAbs Evaluation

Fingerpick microsample-based dried blood spot (DBS) sampling for NAbs determination was developed according to the protocol that we established for monoclonal antibody analysis [16]. This study first evaluated the most appropriate buffer for NAb detection using WT-R001 as the calibrator. First, WT-R001 (0 and 8 μg/mL) were spiked in a mixture of whole blood from healthy donors. Similarly, 40 μL of spiked blood was spotted on filter papers and air-dried for 1 h, followed by being punched out into a 6 mm diameter disc (approximately 10 μL of blood) and immersed into 240 μL of extraction buffer, resulting in a 25-fold predilution. In this study, ten different buffers were tested, including SuperBlock T20 buffer, SuperBlock buffer, SD buffer, high-salt SD buffer, PBS + 0.1% Tween 80, PBS + 0.1% Tween 20, PBS + 0.05% Tween 20, PBS + 0.5% BSA, PBS + 0.05% Tween 20 + 2% BSA, and PBS buffer. The following treatments with shaking, centrifugation and collection of supernatants were the same as in the former study. WT-R001 concentrations were measured using the FO-BLI NAbs biosensor. The buffer that contributed to the highest extraction efficiency of the two DBS samples was elected. Afterwards, WT-R001 series concentrations of (0–1–2–5–10–20 μg/mL) were tested to carefully evaluate the precision of DBS for NAbs determination as compared to sera.

### 2.6. Description of the Study Cohort

This study builds upon our previous research into developing the FO-BLI biosensor, and was conducted with ethical approval from both the Sir Run Run Shaw (SRRS) Hospital, School of Medicine, Zhejiang University (research 20210706-7), and the Institutional Review Board of Westlake University (20210301BSM001). Informed consent was obtained from all the 15 In-Vx-3 healthy adult donors without a previously reported COVID-19 infection. Paired samples were collected at seven defined time points: Day 0 (immediately prior to receiving dose 3), Day 7 (seven days after dose 3), Day 14, Day 21, Day 29, Day 90, and Day 270. Notably, Day 270 samples were provided by six participants who underwent relaxed epidemic control measures in China. Whole blood was collected via venipuncture into BD Vacutainer^®^ Lithium Heparin tubes and then centrifuged at 2000 rpm for 10 min at 25 °C to obtain the sera. Capillary blood was obtained from a finger prick and absorbed on a protein saver card, with the first blood drop discarded. After air-drying, a DBS was extracted. All samples were subsequently de-identified. Levels of NAbs in paired DBS samples and sera were detected using the FO-BLI NAbs biosensor. Moreover, physiological parameters of all participants were included in this study, such as age, gender, body mass index (BMI), blood type, and the interval between the third and second doses.

### 2.7. Designing the FO-BLI Biosensor for Multiplexed Detection of BAbs in Sera

A simplified version of our previously designed FO-BLI bioassay for multiplexed determination of binding antibodies (BAbs) in sera was developed [6]. The purpose was to remove the use of signal enhancer to maximize the chance of fiber reuse without compromising the sensitivity. In summary, SA fibers were first immersed in SD buffer for 10 min to establish a baseline measurement. Next, the fibers were functionalized with biotinylated WT-RBD to capture WT-specific BAbs in clinical samples. Biotinylated WT-RBD was diluted to 2 µg/mL in SD buffer, and the capture shifts were controlled to achieve approximately 1.2 nm. The detection of BAbs was carried out by directly dipping the functionalized fibers into clinical sera, which were first diluted by a factor of 40 to ensure an optimal signal-to-noise ratio, using the high-salt SD buffer. Healthy control sera from six vaccine-naïve volunteers were also used as negative controls to determine the LoD. The processes for establishing the BA.4/BA.5-specific BAbs, BF.7-specific BAbs, and XBB.1.5-specific BAbs biosensors were identical.

### 2.8. Defining the Limit of Detection in Both Sera and DBS Samples for NAbs

To define the limit of detection (LoD) of the techniques, we required a pool of healthy control sera that did not contain SARS-CoV-2 antibodies. Therefore, we recruited six healthy adult volunteers who had not received any vaccine for various reasons to donate paired blood. Volunteers were selected if their serum samples were tested negative for SARS-CoV-2 S-ECD, as determined using the Anti-S-ECD BAbs biosensor with signal enhancer [6], which was previously developed. Afterwards, paired sera and DBS samples were collected as healthy control sera and healthy control DBS samples to determine the LoD for sera and for DBS samples, respectively. The same control sera were used to define the LoD for the new version of the FO-BLI BAbs assay described in the following section.

### 2.9. Validation of the FO-BLI NAbs Biosensor Using In Vitro PVNT

The in vitro PVNT was conducted following a protocol published in *Nature Protocols* [17]. Serum samples were serially diluted four-fold, starting from a 1:20 dilution, and then incubated with a SARS-CoV-2 pseudovirus luciferase reporter (GeneScript, SC2087A) at 37 °C for 1 h. The antibody–recombinant virus mixture was then added to Opti-HEK293/ACE2 cells. After 48 h, the cells were harvested and infected cells were lysed. A luciferase chromogenic solution was added, and the luciferase luminescence (RLU) signals were measured using a microplate reader (Thermo, Variokan LUX) after incubation at room temperature for 3–5 min. The percentage of infected cells was normalized to that derived from cells infected with SARS-CoV-2 pseudovirus luciferase reporter in the absence of serum. The neutralization percentage was calculated using Equation (1). ACE2-Fc was used as a positive control. Neutralization capacities were presented as the 50% maximal inhibitory concentration (IC50). The same procedure was applied for the IC50 of three selected serum NAbs against BA.4/BA.5, BF.7, and XBB.1.5. Samples that did not reach 50% inhibition at the dilution of 1:20 were considered non-neutralizing and set at zero for correlation calculation.

(1) $$Inhibition\;\left(\%\right)=-\left(sample\;RLU-blank\;RLU/positive\;control\;RLU-blank\;RLU\right)$$

### 2.10. Machine Learning-Based Prediction of the Antibody Levels

This section presents the implementation of ML-based inhibition prediction on three different parameters: sera NAbs, DBS NAbs and sera BAbs. The process is depicted in Scheme 1, including merging features, preprocessing, regression modeling, and prediction performance evaluation modules. Due to some absences on Day 270 and no inhibitions found after three months, we only considered the first five batches of measurements for ML-based prediction experiments. Additionally, two participants missed one measurement, leaving 13 participants for further study.

Our primary objective was to predict Day 29 inhibition using the first four datasets of Days 0–21. We considered the characteristics of each participant for ML, such as age, gender, blood type, BMI, and days to receive the third dose. Prior to prediction modeling, we applied preprocessing techniques to each feature, including one-hot encoding for gender and blood type features, and normalization of age, BMI, and dose features to a range of 0 to 1. The first four inhibition results remained unchanged. Due to the similar inhibition trends of the three parameters, we combined the five characteristic features with the first four sets of inhibition measurements from each test as input and used the 5th measurement (Day 29) from each test as the target output. Therefore, we extracted three samples from each participant, resulting in a total of 39 (13 × 3) samples. Finally, we constructed a regression-based prediction task with nine input features (4 measurements + 5 characteristics), with the target output as inhibition at Day 29.

To identify the best regression model for prediction, we employed six different models, including two linear models, two polynomial models with varying degrees of accuracy, support vector machine (SVM), and multilayer perceptron (MLP) methods [18]. We evaluated the models’ performances using the root mean square errors (RMSEs) between the predicted and true values. To validate our results, we implemented a leave-one-out validation scheme, where each run left one sample as the validation sample and the remaining samples were used to train the regression model. This resulted in 39 RMSEs, which we divided into three groups according to three different parameters (i.e., 13 RMSEs for each parameter). We then computed the mean RMSE for each parameter. The RMSE for each parameter was calculated as follows:

(2) $$RMSE_{k}=\sum_{i=1}^{N_{k}}\sqrt{{\left({\hat{y}}_{i}-y_{i}\right)}^{2}}/N_{k},\;k\in parameter\;type$$

where, k is any of the 3 test parameters, and $N_{k}$ in this study should be 13 for each type of k. ${\hat{y}}_{i}\;$and $y_{i}$ stand for the predicted target and the true target, respectively.

### 2.11. Statistical Analysis

**Figure 1:**
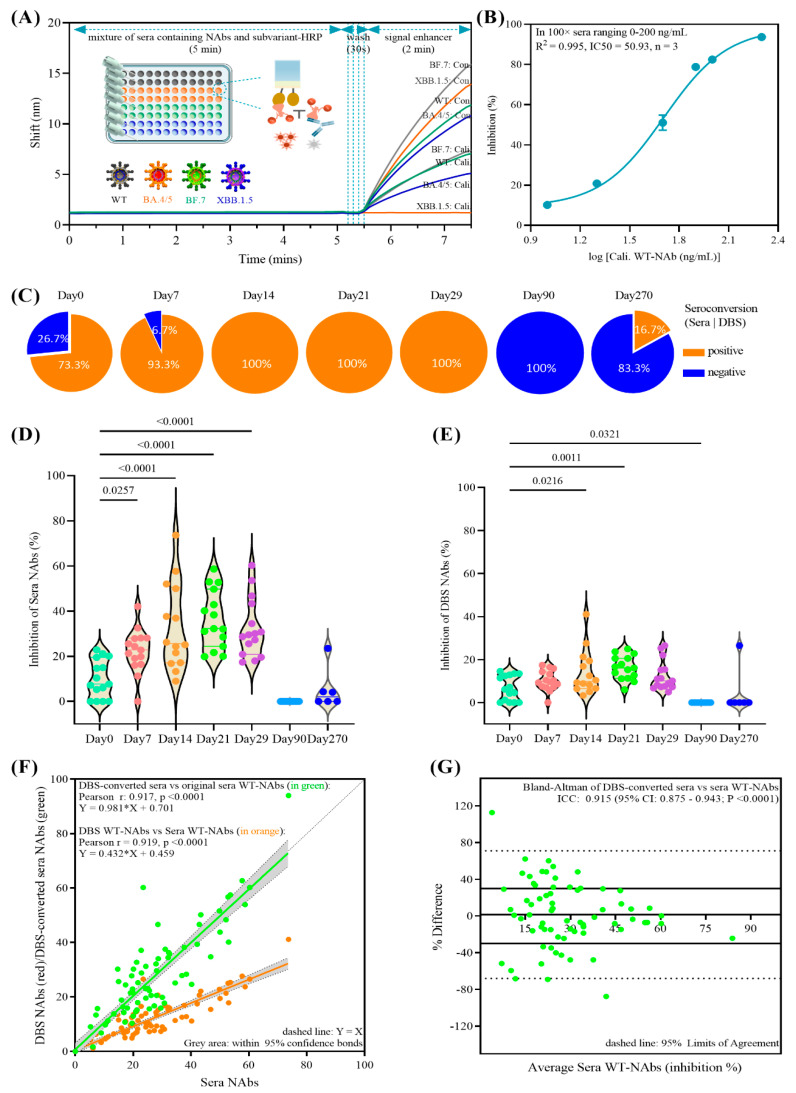
The multiplexed FO-BLI NAbs biosensor and long-term NAbs response towards the wild-type variant in the 15 enrolled In-Vx-3 donors in sera and microsamples. (**A**) Flowchart of the FO-BLI biosensor for multiplexed detection of a calibrator antibody as compared to a control. (**B**) Inhibition curve to detect the calibrator WT-NAbs in both matrices. (**C**) Profiling of WT-NAb levels from Day 0 till Day 270; (**D**) WT-NAbs in sera; (**E**) WT-NAbs in paired microsamples; (**F**) Correlation between sera vs. DBS WT-NAbs (*n* = 94, data in orange) and DBS-converted vs. original sera WT-NAbs (data in green); (**G**) Bland–Altman plot of relative difference between DBS-converted sera vs. original sera WT-NAbs (*n* = 94).

*p* values for differences between groups of sera and DBS NAbs were determined using ANOVA, and nonparametric or mixed methods were used for multiple comparisons and presented in violin plots using GraphPad Prism v9.5.1 (GraphPad Software, San Diego, CA, USA). All the statistical tests were two-tailed, and *p* values of less than 0.05 were considered statistically significant. Inhibition curves against RBD-NAbs and Omicron-NAbs were generated by the FO-BLI NAbs biosensor, and the half maximal inhibitory concentration (IC50) was determined using the “dose-response-inhibitor: log(inhibitor) vs. normalized response—variable slope”. The in vitro PVNT principle in Figure 1 was created using Biorender and ML visualization was drawn using Python 3.8.0. To assess the correlation of inhibition in paired sera and DBS samples, GraphPad Prism 9.5.1 was used, setting any value below the defined LoD as zero. Data are presented as means ± standard deviation (STD), as described in the corresponding figure legends. Linear regression lines were fitted to the data, and their 95% confidence intervals (CI) were calculated using GraphPad Prism. Relative Bland–Altman plots were created using GraphPad Prism, and the intra-class correlation coefficient (ICC) was determined using the “two-way mixed, absolute agreement—single measure test (absolute agreement)” on SPSS Statistics 25 (IBM, Armonk, NY, USA) to investigate mean differences and agreement.

## 3. Results

### 3.1. Multiplexed FO-BLI Biosensor Ability to Detect NAbs in Sera and Microsamples

The ability of the multiplexed FO-BLI biosensor to detect NAbs for WT and Omicron subvariants in 100-fold diluted sera is summarized in Table S1. Compared to its previous version [6], this biosensor used a more environmentally friendly and user-friendly biomaterial 3-Amino-9-ethylcarbazole (AMEC) as a signal enhancer (adopted from our previous study [13]) and the high-salt SD buffer for sample dilution (adopted from another study [14]). Using functionalized fibers, multiple samples or one sample towards multiple subvariants, NAbs were detected within 6.5–7.5 min (Figure 1A). This protocol confirmed that bebtelovimab (BEB), a potent and fully human NAb [15], was ineffective against XBB.1.5, despite its broad neutralizing activity towards Omicron variant lineages BA.4/BA.5 and BF.7 (Figure S1). Also, AMEC-precipitated fibers did not undergo complete regeneration (Figure S2), resulting in a one-fiber-one-sample approach for measuring NAbs.

When evaluating the DBS microsamples’ capacity for NAbs biosensing following a previous procedure [15], five out of the ten listed extraction buffers were discarded due to their high background interference (data not shown), and the following five remained for further consideration: Superblock T20, PBS + T80, PBS + 0.5%BSA, PBS + 0.05%T20, and PBS + 0.05%T20 + 2%BSA. Further experiments proved that PBS + 0.05%T20 + 2%BSA was the most suitable buffer, as the two tested artificial samples had similar performance compared to their sera counterpart (Table S2A,B), showing the highest inhibition (mean of 75.2%, CV of 6.3%, *n* = 4) and the lowest background interference (mean of 2.7%, CV of 8.9%, *n* = 2). Using a series of calibrator WT-NAb concentrations for spiking and extraction, the overall extraction efficiency was determined to be 80.9% with a CV of 5.4% (Table S2C), indicating that 80.9% of calibrator WT-NAbs were extracted from DBS samples. Simultaneously, data confirmed the appropriateness to determine DBS concentrations by interpolating from the inhibition curve of the FO-BLI NAbs biosensor in 100-fold sera (Figure 1B). Moreover, the LoDs for WT-NAb detection in sera and DBS samples were determined to be 4.9% and 0.0%, respectively, using paired samples of six vaccine-naïve healthy donors.

### 3.2. Baseline Characteristics of the Study Cohort

Table 1 shows the baseline characteristics of this cohort study, which consisted of 15 In-Vx-3 healthy donors, 73.3% of whom were female. The mean age was 25.0 y (mean ± standard deviation (STD): 3.6), and the mean body mass index (BMI) was 19.9 (STD: 3.5). All participants received the third dose of COVID-19 vaccine in March 2022, with a mean interval of 7.1 months (STD: 1.10) between dose 2 and dose 3, and 86.7% received the inactivated Sinovac vaccine. Furthermore, six of 15 donors (40%) provided samples nine months after the third dose (i.e., Day 270), which coincided with the period of relaxed pandemic control measures and an outbreak in Hangzhou, China, where all donors resided, resulting in a total of 94 samples included in this study. The consent of the enrolled donors and related approval from the ethics committees were received.

### 3.3. Dynamic Profiling of WT-NAbs in Both Sera and Microsamples

Out of 94 clinical samples, the WT-NAb levels in sera had 5.3–73.7% inhibition, with 25 samples falling below the LoD. Samples that had WT-NAb levels below an LoD of 4.9% were defined as negative and values were set as zero. Particularly, the samples prior to the booster (i.e., Day 0 sera) had a seropositivity of 73.3% (Figure 1C) and a mean WT-Nab level of 7.7% (STD: 8.4%; Figure 1D). We observed a 2.9-fold increase (STD: 9.7%) in the overall levels of sera WT-NAbs one week after vaccination. This increase was slightly higher in Day 14 samples at 3.3-fold (STD: 19.1%) and Day 21 samples at 3.6-fold (STD: 12.8%), which reached a seropositivity of 100%. The increase remained at 3.2-fold (STD: 13.1%) after one month but subsequently gradually declined, and WT-NAbs became undetectable after three months. Notably, the liberalization of epidemic control measures resulted in only one of the six remaining volunteers having a WT-NAb level of 23.5%. Compared to venous sera, DBS microsample sensitively detected WT-NAbs with 100% specificity and identified an identical seropositivity for all samples from Days 0–270 (Figure 1C). Of all 94 samples, DBS sampling also revealed 25 samples that failed the LoD, while the remainder had DBS WT-NAb inhibition levels of 1.2–41.1%. Samples prior to the booster dose (i.e., Day 0 DBS samples) had mean WT-NAbs of 6.2% (STD: 5.8%), and a 1.6-fold increase (STD: 10.4%) in the mean levels of sera WT-NAbs was found on Day 7 (Figure 1E). This increase was relatively stable for Day 14 samples, at 1.4-fold (STD: 10.4%), and Day 21 samples at 1.6-fold (STD: 5.6%). The increase remained at 1.3-fold (STD: 7.0%) after one month and gradually declined to become undetectable after three months. Similarly, WT-NAb were detected in the paired DBS samples of the same volunteer from whom serum antibodies were elicited on Day 270.

Collectively, the Pearson correlation analysis revealed a strong correlation between WT-Nab levels in DBS samples and sera (Pearson r = 0.919, *p* < 0.0001, *n* = 94; Figure 1F, in orange). A linear regression equation ([WT-NAbs]DBS = [WT-NAbs]sera *0.432 + 0.459, R2 = 0.919, *p* < 0.0001) was found to convert the DBS WT-NAb levels to sera levels (i.e., DBS-converted sera). The WT-NAb levels of the DBS-converted sera were not significantly different from the originally obtained sera levels (correlation coefficient (ICC) = 0.917, *p* < 0.0001) (Figure 1F, in green). The Bland–Altman plot revealed an average bias of 1.5 (95% CI: 68–71%) between the DBS-converted and originally-obtained sera WT-NAb levels, where 74% of samples were within 30% of their mean (Figure 1G). These results satisfy the criteria of the FDA and EMA, which require at least 67% of clinical samples to be within 30% of their mean of difference.

### 3.4. Multiplexed Profiling of NAbs towards Omicron BA.4/5, BF.7 and XBB.1.5

The study further assessed the extent to which the newest omicron lineages could evade neutralization by WT-elicited antibodies. At the start of the study WT coronavirus was predominant; however, as of early 2023, BA.4/BA.5, BF.7 and XBB.1.5 lineages have become more prevalent. The extended duration of the study has rendered DBS extractions collected in the early stages less suitable for antibody evaluation. Therefore, sera samples were used for this evaluation. Results showed that nine (9.6%) out of 94 sera exhibited positive NAb activity towards BA.4/BA.5, five (5.3%) were positive towards BF.7, and one (1.0%) was positive towards XBB.1.5 (Table S3). These data suggest considerable but incomplete escape of BA.4/BA.5, BF.7, and XBB.1.5 from the WT-NAbs elicited by the third dose. Notably, of the six Day 270 sera collected after the relaxed epidemic control measures that caused a significant Omicron outbreak, three sera simultaneously developed NAbs towards BA.4/BA.5 (50%) and BF.7 (50%), and one serum (of donor no. 13) reacted towards XBB.1.5. This result shows the broad-spectrum characteristics of this serum NAb towards Omicron subvariants.

### 3.5. Validation of the FO-BLI NAbs Biosensor Using Clinically Validated PVNT

**Figure 2:**
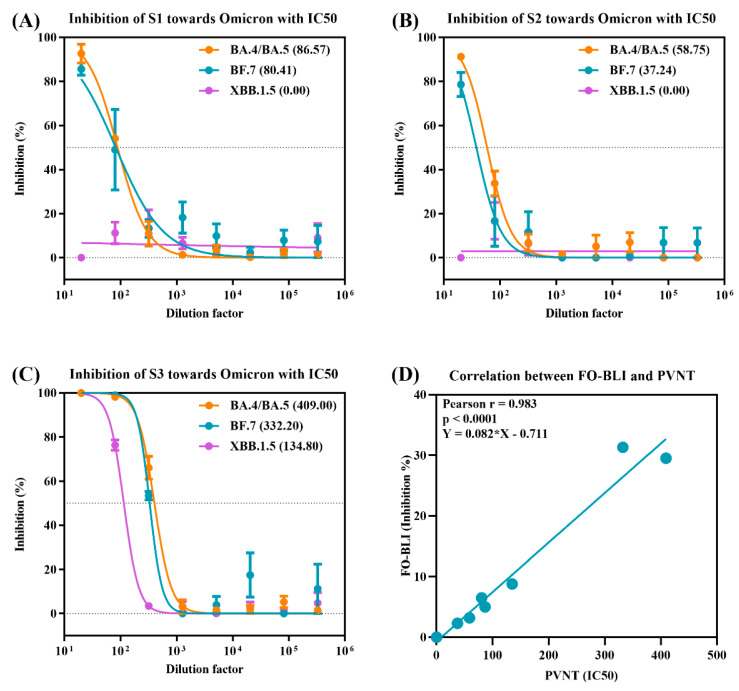
Validation of the FO-BLI NAbs biosensor using a PVNT. (**A**–**C**) Inhibition percentage profiles with IC50 values of three serum NAbs (S1–S3) towards omicron BA.4/BA.5, BF.7 and XBB.1.5, as measured using the clinically validated PVNT. (**D**) Correlation of the measurements between FO-BLI and PVNT.

It is critical to understand whether the NAb levels obtained through this multiplexed NAbs FO-BLI biosensor precisely reflect the neutralization capacities observed in standard assays. To investigate this issue, a commercially available and clinically validated PVNT was performed to determine the IC50s of three sera towards the latest subvariants. Figure 2A–C shows the inhibition percentage profiles of three sera NAbs and their IC50 values determined by the PVNT. These profiles demonstrate consistent positivity and negativity towards each subvariant. Comparison of the IC50 values determined by the PVNT and NAb levels determined by the FO-BLI NAbs biosensor reveals a strong correlation (Figure 2D; Table S4) with a Pearson r of 0.983 (*p* < 0.0001) and a linear regression of Y = 0.082 × X − 0.711. Of note, S1 is the serum collected from donor no. 11 on Day 14, S2 is the serum collected from donor no. 10 on Day 10, and S3 is the serum from donor no. 13 on Day 270, as shown in Table S3.

### 3.6. Multiplexed Biosensing of Sera BAbs towards Both WT and Variants

**Figure 3:**
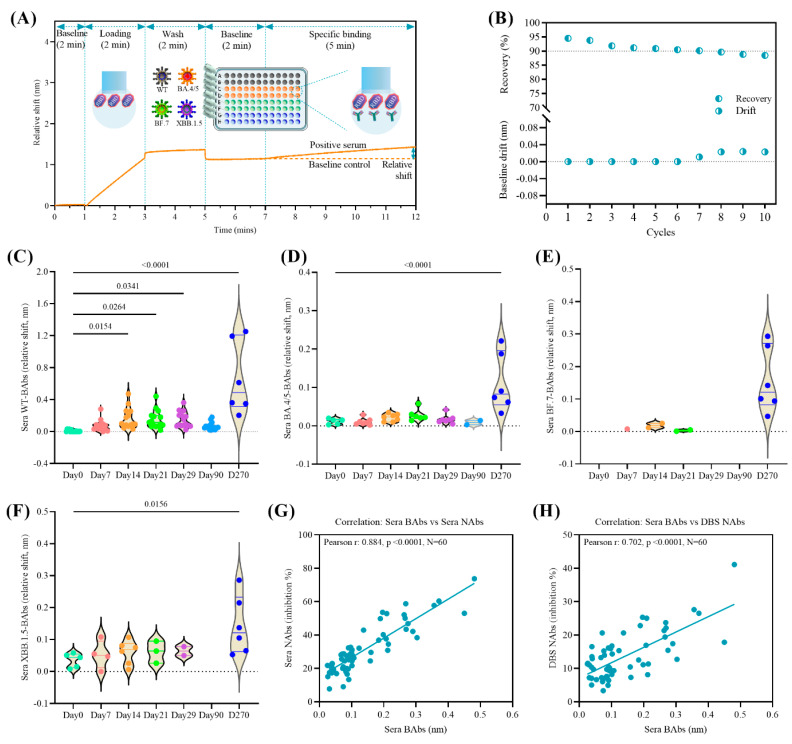
Performance of the multiplexed FO-BLI BAbs biosensor and long-term BAbs response in the 15 enrolled In-Vx-3 donors. (**A**) Flowchart of the multiplexed FO-BLI BAbs biosensor towards the WT strain and Omicron subvariants in 40-fold diluted sera. (**B**) Regeneration capability of the FO-BLI BAbs biosensor using Glycine 10 mM pH 2.0 as the solution. Profiling of the sera BAb levels towards (**C**) the WT strain, (**D**) BA.4/BA.5, (**E**) BF.7, and (**F**) XBB.1.5 on Days 0–270 using the FO-BLI BAbs biosensor. Correlation between (**G**) sera BAb versus sera NAb levels and (**H**) sera BAb versus DBS NAb levels for Days 0–29 samples.

A simplified version of the multiplexed BAbs FO-BLI biosensor was developed to obtain a more comprehensive profile of the antibody response of all donors (Figure 3A). The signal-enhancer-free sensor is regenerable up to six times when using 10 mM Glycine pH 2.0 as the regeneration solution, with a recovery of above 90.5% and a zero baseline drift (Figure 3B). Pre-functionalized probes allowed the detection of BAbs in 40-fold diluted sera in 7.0 min (Table S5). Moreover, the FO-BLI BAbs biosensor demonstrated excellent reproducibility when tested on seven individual sera, with a variation of less than 12% during two separate runs (Table S6). These results support the cost-effectiveness, robustness, and reliability of the BAbs biosensor for use in clinical samples. Furthermore, the FO-BLI BAbs biosensor provided additional evidence that the BEB antibody cannot bind to XBB.1.5, despite its activity against previous subvariants (Figure S3).

For the study cohort, sera levels of BAbs for the WT strains BA.4/BA.5, BF.7 and XBB.1.5 were profiled. The relative shifts of WT-BAbs in 94 sera exhibited significant variation, and nine samples fell below the LoD (see Figure 3C). Moreover, we observed a notable 8.5-fold increase in overall levels of sera WT-BAbs one week after the vaccination. This increase was particularly striking on Day 14, when the levels had increased by 20.7-fold, while subsequently remaining stable on Day 21 (at 19.1-fold) and Day 29 (at 18.5-fold). However, three months after the vaccination, the increase in WT-BAbs dropped to 8.9-fold. Notably, following the liberalization of epidemic control measures, the increase in levels of WT-BAbs climbed sharply to 82.9-fold on Day 270, based on the results from six donor samples. High BAb levels were developed for the three Omicron lineages with sera positivities of 42.5%, 11.7% and 27.6% for BA.4/BA.5 (Figure 3D), BF.7 (Figure 3E), and XBB.1.5 (Figure 3F), respectively. Notably, all six Day 270 sera collected during the Omicron outbreak produced BAbs for the three subvariants. Further analysis of the measurements in the first month revealed a clear correlation between the relative shifts of sera WT-BAbs and inhibition percentages of WT-NAbs in both sera (Pearson r = 0.884, Figure 3G) and DBS samples (Pearson r = 0.702, Figure 3H).

### 3.7. Machine Learning-Based Modeling to Predict Antibody Levels over Time

**Figure 4:**
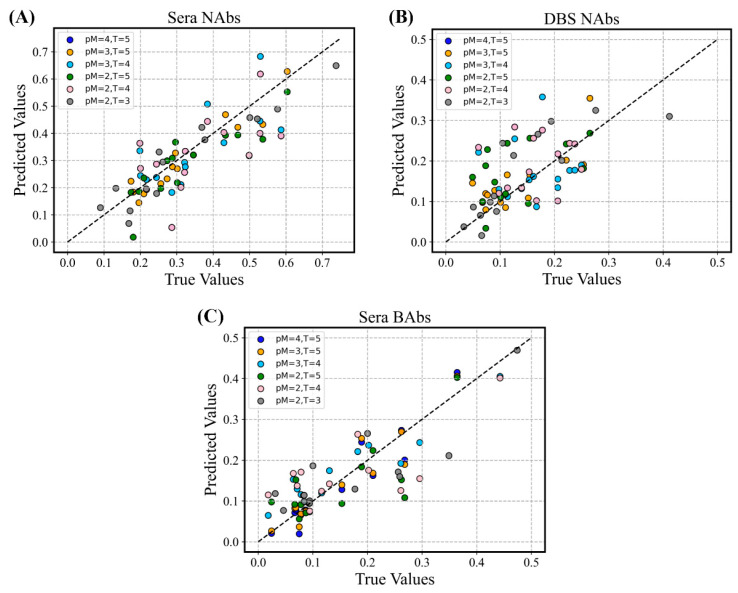
Visualization of the three different parameters. Profiles of three main parameters: predicted (**A**) sera NAbs, (**B**) DBS NAbs and (**C**) sera BAbs versus the true values.

This section aims to investigate the feasibility of predicting the antibody response using ML techniques. Table S7 shows the initial performance of sera NAbs, DBS NAbs and sera BAbs obtained by six regression models to predict antibody levels at Day 29. The lasso linear model outperformed all the other five models with a less than 5% RMSE and was therefore selected for further prediction tasks. We then continued to investigate whether we could make a longer-term prediction based on relatively limited data. Thus, we predicted the measurements at a given time point based on previous measurements (i.e., *pM*), where $T\in \left\{2,\;3,\;4\right\}$, $pM\in \left\{1,\;2,\;3\right\}$. Table 2 shows the prediction performance of a series of *T* and *pM* sets using the lasso linear model, which accurately predicted the measurements with an RMSE within 5% at several timepoints. Regarding the sera NAbs, data from Days 0, 7 and 14 could predict the data on Day 29 with similar accuracy (RMSE of 3.7%). Similar accuracy was also observed for the DBS NAbs. Interestingly, regarding DBS NAbs, Day 0 data could precisely predict the data of Day 7 (RMSE of 4.9%), and the data of Days 0–7 could predict the Day 14 data with high accuracy (RMSE of 5.3%). Compared with the sera NAbs, DBS NAbs could be predicted with high precision at two more time points. Regarding the sera BAbs, more accurate data were predicted using this lasso linear model. For example, data from Day 14 and onwards were successively obtained from the data from Days 0–7. Figure 4A–C displays visual representations of the three parameters, where the predicted values are plotted against the actual measured values.

## 4. Discussion

This study integrated the multiplexed FO-BLI biosensor with microsampling for rapid and dynamic profiling of NAbs towards both the WT strain and Omicron COVID-19 variants in 15 In-Vx-3 healthy donors, with a follow-up period of nine months. We collected 94 paired samples and found that the levels of WT-NAbs from DBS microsamples were consistent with those obtained in venipuncture sera, with a Pearson correlation coefficient of 0.919 and a specificity of 100%. A linear regression was built to support a simple interchange of the data, proving the accuracy and reliability of DBS in evaluating NAbs under optimized conditions. Microsampling additionally proved its potential to meet the global demand for both large-scale and personalized management [19]. Moreover, validation of the FO-BLI NAb biosensor using nine individual sera of three vaccinated donors showed its excellent accuracy in assessing the neutralizing capabilities of NAbs compared to a standard PVNT (Pearson r correlation 0.983). This further confirms the clinical utility of the biosensor and the reliability of the data produced to build ML models. To obtain a richer profile of antibody response, we simplified the FO-BLI BAbs biosensor to regenerate a single fiber for multiple uses and enable high-throughput screening at a lower cost.

With our technologies, the data showed a 3.3-fold increase in sera WT-NAb levels after 0.5 months and a 3.2-fold increase after one month following the booster-inactivated vaccines compared to the levels at Day 0. A similar trend was found with the DBS WT-NAb levels (1.4-fold and 1.3-fold). However, the increase was sharper in WT-BAb levels during the same period (20.7-fold and 18.5-fold). Despite the undetectable levels in WT-NAbs at three months, all sera developed good amounts of WT-BAbs (8.5-fold increase). After the epidemic control was loosened, the volunteers produced the elicited antibodies of both types, which were possibly restored by the Omicron outbreak. The antibody response towards Omicron, including its subvariants BA.4/BA.5, BF.7 and XBB.1.5, has shown extensive but incomplete escape from the WT-NAbs elicited by the booster. However, we identified a broad-spectrum serum NAb (Day 270 sample of donor 13) that could neutralize all of the latest Omicron subvariants, and which offers a potent NAb candidate. Similarly, all six donors available on Day 270 during the Omicron outbreak developed more NAbs and BAbs towards all three subvariants. Our data and others provide compelling evidence that a homologous booster of inactivated vaccine can enhance antibody response, although its advantages against Omicron variants are limited [20]. Thus, there is a need in receiving a heterologous or Omicron-specific booster vaccines to better address the challenges posed by these variants [21,22].

ML is poised to play a pivotal role in driving deep evidence-based medicine [23]. Although numerous clinical studies have employed ML to predict antibody responses following vaccinations, most have focused on predicting the probability of seroconversion at a single timepoint instead of predicting precise levels of antibody levels and their temporal dynamics in individuals [10,11,24,25,26,27]. Additional studies have investigated the prediction of neutralization titers to multiple Omicron variants after breakthrough infections [28]. In this study, we bridged this gap by developing a model that can accurately predict the changes in antibody response at multiple timepoints based on previous measurements. Of note, our study found that simple linear models performed better than more complex ML algorithms, likely due to the limited size of our dataset. Moreover, we chose not to apply the model to predict antibody response toward Omicron, as the inactivated vaccines primarily target the ancestral strain, and the complex nature of emerging variants presents challenges for accurate modeling. This model aims to provide a valuable framework for predicting the booster-induced antibody levels in healthy individuals, eventually enhancing the precision of vaccination strategies tailored to this specific population.

Our study does have several limitations. Firstly, the study cohort’s sample size is relatively small, which restricted our ability to fully comprehend the antibody response to the booster-inactivated vaccine and construct a precise prediction model. To enhance the model’s accuracy, further investigations are necessary, involving larger datasets that encompass a balanced representation across genders and age groups. Additionally, incorporating lifestyle-associated parameters extracted from microsamples would bolster these efforts [29]. Secondly, the microsamples collected during the early stages did not allow for the evaluation of NAbs against Omicron due to the extended duration of our study. This highlights the imperative need to enhance the storage stability of microsamples for prolonged research studies. Thirdly, despite the robust correlation observed between the FO-BLI NAbs biosensor and the pseudovirus-based neutralization assay, validating the findings using a real-virus neutralization assay is of paramount importance in a clinical context.

## 5. Conclusions

Collectively, we have developed two advanced FO-BLI biosensors that enable the rapid and multiplexed detection of NAbs and BAbs against the wild-type COVID-19 strain and Omicron sublineages. The FO-BLI NAbs biosensor exhibits a strong correlation with a standard PVNT, demonstrating its clinical utility for real-life application. The regenerable capability of the FO-BLI BAbs biosensor makes it a promising and cost-effective tool for high-throughput screening. Moreover, our use of microsampling as a simple yet reliable method for dynamically profiling NAbs has opened new avenues for personalized antibody response monitoring. Integrating microsamples with the FO-BLI biosensor enables individuals to receive their test results within hours of self-sampling. The accurate ML model obtained for predicting antibody levels at multiple timepoints in the study cohort not only affirms the biosensor’s reliability but also underscores its potential to expedite decision-making for personalized vaccination strategies. Additionally, the system’s innate adaptability means that it typically requires just one week to be reconfigured for a new pathogen in a pre-clinical setting with access to specific antibodies. This positions it favorably for addressing various disease prevention priorities and assessing novel vaccine candidates.

## Figures and Tables

**Table 1:** Baseline characteristics of the study cohort. Overview characteristics of the healthy donors who received the third dose of COVID-19 vaccine with a follow-up duration of up to nine months.

No.	Sex	Age Range	Blood Type	BMI	Dose 1, 2, and 3 Vaccines	Interval (Month)	Last Time Point
1	Female	21–25	A	19.9	Sinovac	8.3	Day 90
2	Male	21–25	O	28.7	Sinopharm	6.4	Day 90
3	Female	26–30	AB	19.8	Sinovac	6.9	Day 270
4	Female	21–25	B	18.6	Sinovac	7.0	Day 90
5	Female	31–35	O	24.7	Sinovac	5.8	Day 90
6	Female	21–25	A	18.1	Sinovac	6.8	Day 270
7	Female	21–25	O	16.8	Sinovac	6.1	Day 270
8	Male	31–35	B	21.1	Sinovac	7.9	Day 90
9	Female	21–25	O	19.1	Sinovac	6.3	Day 90
10	Female	26–30	O	16.6	Sinovac	7.2	Day 270
11	Female	21–25	O	20.6	Sinopharm	6.5	Day 90
12	Female	26–30	O	19.9	Sinovac	9.6	Day 90
13	Female	26–30	O	20.9	Sinovac	8.0	Day 270
14	Male	26–30	A	20.1	Sinovac	9.0	Day 270
15	Male	21–25	AB	27.5	Sinovac	7.7	Day 90

**Table 2:** ML-modeling-based prediction. Prediction performance of the measurement (measurements at a given time point) based on previous measurements (pM). The prediction was made using the lasso linear model for three parameters.

Input of Previous Measurements (pM), Output at a Given Time Point (T)	Sera NAbs (RMSE)	DBS NAbs (RMSE)	Sera BAbs (RMSE)
pM=4,T=5	3.1%	4.2%	2.7%
pM=3,T=5	3.7%	4.0%	2.6%
pM=3,T=4	9.0%	6.3%	4.2%
pM=2,T=5	6.0%	6.5%	4.7%
pM=2,T=4	9.9%	6.1%	6.5%
pM=2,T=3	6.6%	5.3%	5.3%
pM=1,T=5	14.4%	10.1%	7.8%
pM=1,T=4	17.3%	10.1%	10.2%
pM=1,T=3	15.9%	11.2%	9.5%
pM=1,T=2	9.4%	4.9%	7.1%

## Data Availability

Data will be available on request.

## Supplementary Materials

The following supporting information can be downloaded at https://www.mdpi.com/article/10.3390/vaccines12040352/s1. Figure S1: Inhibition of the calibrator BEB towards BA.4/BA.5, BF.7 and XBB.1.5 by the FO-BLI NAbs biosensor; Figure S2: Three groups of fibers with controlled signals ranging from 0.5 nm to 40 nm were prepared to evaluate the effect of high-purity ethanol on cleaning and regenerating the fibers. Data showed that no fiber can be fully cleaned for reuse under the conditions tested; Figure S3: Binding activities of BEB towards BA.4/BA.5, BF.7 and XBB.1.5, respectively, showing no interaction between BEB and the latest XBB.1.5. Table S1: The FO-BLI biosensor for multiplexed biosensing of NAbs towards WT and Omicron in both sera and DBS using AMEC as the signal enhancer; Table S2: Summary of performance of DBS microsample in (A) positive and (B) negative samples in five pre-selected extraction buffers, and (C) extraction efficiency of series of DBS spiked samples in the selected buffer. CV—coefficient of variation; Table S3: Seropositivity towards the latest omicron subvariants of all 94 sera samples at all timepoints; Table S4: Summary of the IC50 defined by the PVNT of three serum NAbs towards Omicron subvariants; Table S5: FO-BLI multiplexed biosensing of BAbs towards both the WT strain and Omicron in sera; Table S6: Reproducibility of the FO-BLI BAbs biosensor for detecting WT-BAbs in seven individual serum samples from seven individual donors on Day 21; Table S7: Accuracy of six regression models to predict the inhibition of Day 29 samples. The three parameters as desired were predicted based on four previous measurements of Days 0–21 and compared to the measured inhibition data.
