# Supplementary material for: Dynamic Profiling and Prediction of Antibody Response to SARS-CoV-2 Booster-Inactivated Vaccines by Microsample-Driven Biosensor and Machine Learning
Source: Vaccines (Basel). 2024 Mar 25;12(4):352. doi: 10.3390/vaccines12040352 (PMC11054503; doi:10.3390/vaccines12040352)
Supplement: Supplementary file 1 [file vaccines-12-00352-s001.zip › vaccines-2875504-supplementary.pdf]

Supplementary Figures

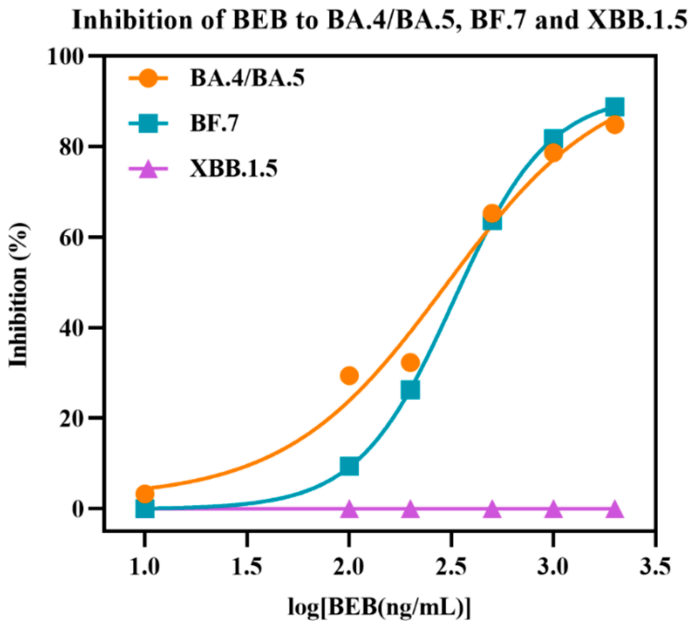

Figure S1. Inhibition of the calibrator BEB towards BA.4/BA.5, BF.7 and XBB.1.5 by the FO-BLI NAbs biosensor.

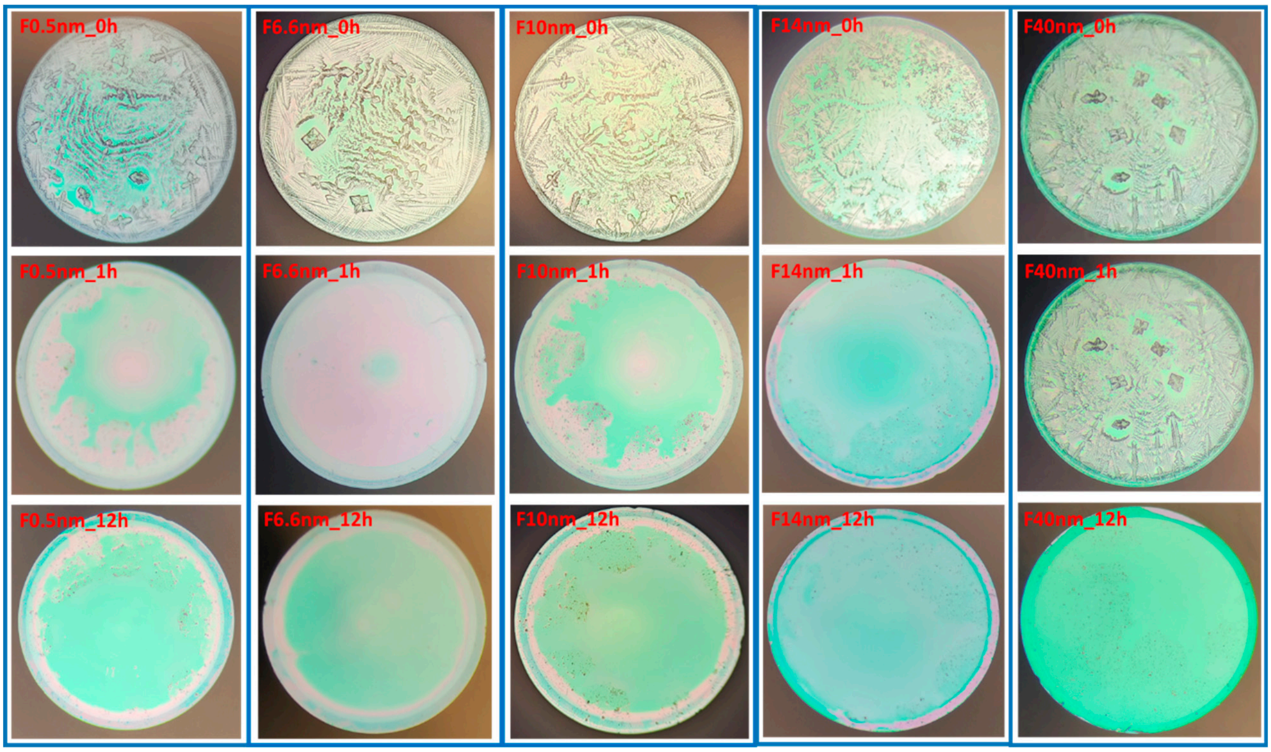

Figure S2. Three groups of fibers with controlled signals ranging from 0.5 nm to 40 nm were prepared to evaluate the effect of high-purity ethanol on cleaning and regenerating the fibers. Data showed that no fiber can be fully cleaned for reuse under the condition tested.

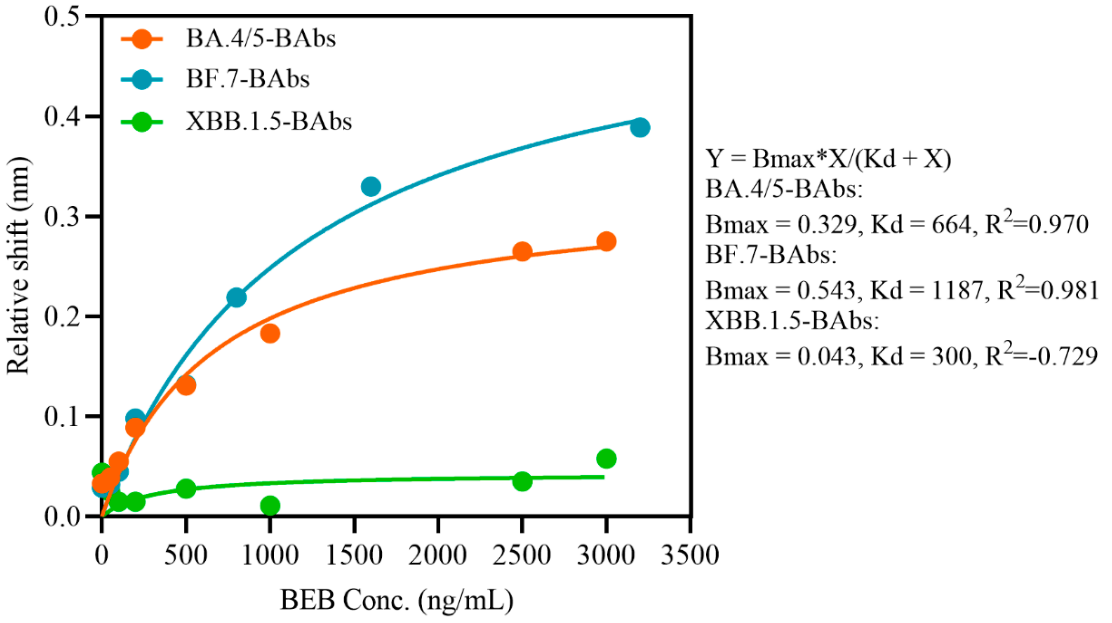

Figure S3. Binding activities of BEB towards BA.4/BA.5, BF.7 and XBB.1.5, respectively, showing no interaction between BEB and the latest XBB.1.5.

Supplementary Tables

Table S1. The FO-BLI biosensor for multiplexed biosensing of NAbs Towards WT and Omicron in Both Sera and DBS Using AMEC as the signal enhancer.

Main Items	Assay conditions[1]	Anti-WT RBD NAbs	Anti-Omicron RBD NAbs (BA.4/BA.5, BF.7, XBB.1.5)
**Capture Loading**	Probe	SA sensor	SA sensor
	**Capture protein (conc.)**[1]	**hACE2-B** (5 ug/mL)	**hACE2-B** (5 ug/mL)
	Capture buffer[2]	SD	SD
	Capture shift	1.0 nm	1.0 nm
	Washing	30s in SD	30s in SD
**Sample Processing**	**Sample type**	**Sera, DBS**	**Sera, DBS**
	Sample volume	5 μL	5 μL
	Sample dilution	1/100 [DBS: 1/25 pre-diluted]	1/100 [DBS: 1/25 pre-diluted]
	Sample buffer	High-salt SD	High-salt SD
**Competitive Bind-ing**	**Protein RBD conjugate**	**WT-HRP**	**Omi.-HRP**
	Protein-HRP conc.	WT-HRP	BA.4/BA.5-HRP: 1/3000<br>BF.7-HRP: 1/4000<br>XBB.1.5-HRP: 1/1000
	Dilution buffer	High-salt SD	High-salt SD
	Competitive binding	100 uL sample + 100 uL WT-HRP	100 uL sample + 100 uL Omi.-HRP
	Binding time	5 min	5 min
	Washing	30 s in high-salt SD	30 s in high-salt SD
**Amplify Signals**	**Enhancer**	**AMEC**	**AMEC**
	Enhancing time	1 - 2 min	1 - 2 min
**Assay Properties**	**Calibrators**	**WT-R001**	**BEB (not for XBB.1.5-NAbs)**
	Detection range	10 - 200 ng/mL	10 - 2000 ng/mL
	Signal unit	Relative inhibition percentage	Relative inhibition percentage
	LoD for sera	WT-NAbs: 4.9% inhibition	Omi.-NAbs: 0.0% inhibition
	LoD for DBS	WT-NAbs: 0.0% inhibition	Omi.-NAbs: 0.0% inhibition
	**Detection time**[3]	**6.5 - 7.5 min using pre-functionalized fibers**	
	Fiber regeneration	Not allowed	Not allowed

[1] All the conjugated protein-HRP obtained an initial concentration of 0.5 mg/mL before use.
[2] The use of SD buffer to dilute hACE2-B reduced the shift drift over time.
[3] NAbs detection can be shortened to 6.5 min by slightly increasing the concentration of protein-HRP conjugate while decreasing the enhancer time to 1 min.
[4] AMEC, 3-Amino-9-ethylcarbazole; BEB, Bebtelovimab; hACE2-B, biotinylated human ACE2; Omi.-HRP, HRP conuagted with omicron RBD protein; SA, Streptavidin; WT, wide type.

Table S2. Summary of performance of DBS microsample (A) positive and (B) negative samples in five pre-selected extraction buffers and (C) extraction efficiency of series of DBS spiked samples in the selected buffer. CV, Coefficient of Variation.

(A) Inhibition percentages of one positive sample with spiked concentration at 8 ug/mL (i.e., DBS 8) in five pre-selected buffers compared to their serum counterpart (i.e., NAb 8)

Sample	Extraction buffer	Inhi.1	Inhi.2	Inhi.3	Inhi.4	Inhi.5	Mean	STD	CV
**NAb 8**	100× sera	75.5%	74.0%	72.0%	78.8%	N/A	75.1%	2.9%	3.8%
**DBS 8**	Superblock T20	51.9%	51.8%	50.2%	69.5%	67.9%	58.2%	9.6%	16.4%
	PBS+T80	64.8%	64.9%	62.6%	73.1%	72.6%	67.6%	4.9%	7.2%
	PBS+ 0.5%BSA	72.6%	64.8%	67.0%	77.6%	74.5%	71.3%	5.3%	7.4%
	PBS+ 0.05%T20	64.6%	73.2%	68.0%	79.0%	77.1%	72.4%	6.1%	8.4%
	PBS+0.05%T20+2% BSA	70.9%	71.7%	77.6%	80.8%	N/A	75.2%	4.8%	6.3%

(B) Inhibition percentages of one negative sample (i.e., DBS 0) in the five pre-selected buffers compared to their serum counterpart (i.e., NAbs 0)

Sample	Extraction Buffer	Inhi.1	Inhi.2	Mean	STD	CV
NAb 0	100× sera (as control)	1.1%	-1.1%	0.0%	1.5%	N/A
DBS 0	Superblock T20	-10.7%	-2.3%	-6.5%	5.9%	-91.2%
	PBS+T80	-0.6%	-1.1%	-0.9%	0.4%	-47.4%
	PBS+0.5%BSA	-0.4%	-1.0%	-0.7%	0.4%	-59.6%
	PBS+0.05%T20	2.8%	1.5%	2.1%	0.9%	43.3%
	PBS+0.05%T20+2%BSA	2.9%	2.5%	2.7%	0.2%	8.9%

(C) Extraction efficiency of the DBS microsample using spiked WT-R001 concentrations in the selected extraction buffer of PBS+0.05%T20+2%BSA. Measured concentrations were interpolated from the inhibition curve of the AMEC-based FO-BLI NAbs biosensor in sera as shown in Fig. 1B.

Spiked (ug/mL)	Measured Conc. in DBS extracts (ug/mL, n = 3-6)									Extraction Efficiency
	Test 1	Test 2	Test 3	Test 4	Test 5	Test 6	Mean	STD	CV	
**0**	0.00	0.00	0.00	0.00	/	/	0.00	0.00	N/A	N/A
**2.5**	/	1.98	2.15	2.21	/	/	2.11	0.12	5.7%	84.4%
**4**	3.46	2.45	3.02	3.28	3.60	3.60	3.23	0..44	13.7%	80.9%
**8**	5.51	4.84	6.07	7.08	7.30	6.70	6.25	0.95	15.3%	78.1%
**16**	14.30	12.62	14.38	14.62	14.20	14.50	14.10	0.74	5.3%	88.2%
Overall extraction efficiency										**80.9%**
STD										**4.4%**
CV										**5.4%**

Table S3. Seropositivity towards the latest omicron subvariants of all 94 sera samples at all timepoints.

Sample ID		NAbs (in inhibition percentage)			
Time point	Donor Nr.	WT	BA.4/BA.5	BF.7	XBB.1.5
Day 7	13	28.1%	8.7%	0.0%	0.0%
Day 14	10	73.7%	3.2%	2.3%	0.0%
	11	57.7%	5.0%	6.5%	0.0%
	13	36.9%	9.6%	0.0%	0.0%
Day 21	13	42.9%	8.3%	0.0%	0.0%
Day 29	10	60.3%	6.4%	0.0%	0.0%
Day 270	6	4.1%	3.8%	9.5%	0.0%
	13	23.5%	29.5%	31.3%	8.8%

14	4.3%	3.0%	14.9%	0.0%

Table S4. Summary of the IC50 defined by the PVNT of three sera NAbs towards Omicron subvariants.

Sample	Subvariants	PVNT (IC50)	FO-BLI (Inhibition)
S1	BA.4/BA.5	86.57	5.0%
	BF.7	80.41	6.5%
	XBB.1.5	0.0%	0.0%
S2	BA.4/BA.5	58.75	3.2%
	BF.7	37.24	2.3%
	XBB.1.5	0.0%	0.0%
S3	BA.4/BA.5	409	29.5%
	BF.7	332.2	31.3%
	XBB.1.5	134.8	8.8%

Table S5. FO-BLI multiplexed biosensing of BAbs towards both WT strain and Omicron in sera.

Main Items	Assay conditions	Anti-WT RBD BAbs	Anti-Omicron RBD BAbs		
Capture Loading	Probe	SA sensor	SA sensor	SA sensor	SA sensor
	**Capture (conc.)**[1]	**RBD-B** (2 ug/mL)	**BA.4/BA.5-B** (2 ug/mL)	**BF.7-B** (2 ug/mL)	**XBB.1.5-B** (2 ug/mL)
	Capture buffer[2]	SD	SD	SD	SD
	Capture shift	1.2 nm	1.2 nm	1.2 nm	1.2 nm
	Washing	30s in SD	30s in SD	30s in SD	30s in SD
Baseline	**Baseline**	**HCS**	**HCS**	**HCS**	**HCS**
	Matrix dilution	1/40	1/40	1/40	1/40
	Baseline time	2 min	2 min	2 min	2 min
Sample Binding	**Sample type**	**serum**	**serum**	**serum**	**serum**
	Sample volume	5 μL	5 μL	5 μL	5 μL
	Sample dilution	1/40	1/40	1/40	1/40
	Sample buffer	HS-SD	HS-SD	HS-SD	HS-SD
	Sample time	5 min	5 min	5 min	5 min
Assay Properties	**Calibrator**[3]	**BEB**	**BEB**	**BEB**	**N/A**
	Detection range	50–3000 ng/mL	50-3000 ng/mL	50-3000 ng/mL	N/A
	Signal unit	relative binding shift	relative binding shift	relative binding shift	relative binding shift
	Cut-off D	0.007 nm	0.014 nm	0.015 nm	0.072 nm
	**Detection time**	**7.0 min, using the pre-functionalized probe**			
	Fiber regeneration	Allowed			

[1] All the conjugated protein obtained an initial concentration of 1.0 mg/mL before use.
[2] The use of SD buffer to dilute Protein-B reduced the shift drift over time.
[3] Day270 serum of donor 13 may serve as a universal BAbs calibrator for both WT and omicron variants.
[4] BEB, Bebtelovimab; HCS, healthy control serum; HS-SD, high-salt SD buffer.

Table S6. Reproducibility of the FO-BLI BAbs biosensor for detecting WT-BAbs in seven individual serum samples from seven individual donors on Day21.

Donor Nr.	Test 1 (nm)	Test 2 (nm)	Mean (nm)	STD (nm)	CV
3	0.027	0.023	0.025	0.003	11%
9	0.092	0.079	0.085	0.009	10%
10	0.439	0.460	0.450	0.015	3%

11	0.302	0.303	0.303	0.001	0%
13	0.137	0.137	0.137	0.000	0%
14	0.084	0.096	0.090	0.009	10%
15	0.077	0.065	0.071	0.009	12%

Table S7. Accuracy of six regression models to predict the inhibition of Day 29 samples. The three parameters as desired were predicted based on four previous measurements of Days 0–21 and compared to the measured inhibition data.

Regression Model	Sera NAbs (RMSE)	DBS NAbs (RMSE)	Sera BAbs (RMSE)
Linear model – Linear regression	3.1%	4.3%	2.9%
Linear model – Lasso	3.1%	4.2%	2.7%
Polynomial model – 2 degrees	4.0%	4.8%	3.4%
Polynomial model – 3 degrees	8.1%	4.6%	4.9%
Support vector machine (SVM)	11.0%	7.0%	9.7%
Multilayer perceptron (MLP)	12.0%	7.8%	9.3%
